# Peripheral Neuropathy Caused by Paclitaxel and Docetaxel: An Evaluation and Comparison of Symptoms

**Published:** 2013-07-01

**Authors:** Cindy Tofthagen*, R. Denise McAllister, Constance Visovsky

**Affiliations:** From University of South Florida, College of Nursing, Tampa, Florida; *postdoctoral fellow at University of Massachussets Boston and Dana-Farber Cancer Institute

## Abstract

The purpose of this study was to explore the prevalence, severity, distress, and timing of neuropathic symptoms in cancer patients receiving taxanes and to explore neuropathy-related interference with activities. In this descriptive, cross-sectional study, 68 adult outpatients receiving paclitaxel (n = 36) and docetaxel (n = 32) completed the Chemotherapy Induced Peripheral Neuropathy Assessment Tool and a demographic questionnaire. Muscle or joint aches were the most prevalent symptom. Muscle or joint aches were also the most severe and distressing symptom in persons receiving paclitaxel. Participants receiving paclitaxel reported that neuropathic symptoms interfered with a mean of 7.3 (standard deviation [SD] = 4.1) of 14 activities. Nerve pain was the most severe and distressing symptom in persons receiving docetaxel. Participants receiving docetaxel reported that neuropathic symptoms interfered with a mean of 7.1 (SD = 4.1) of 14 activities. Numbness in the feet was the most frequent or constant symptom in persons receiving paclitaxel or docetaxel. Patients receiving paclitaxel and docetaxel experienced similar symptoms of peripheral neuropathy and interference with activities. Continued focus on treatment of painful neuropathy including myalgias and arthralgias is needed.

Advances in chemotherapy have resulted in improvements in disease-free survival and overall survival for persons with cancer, and these improved outcomes are often associated the use of taxane-based regimens (Jones et al., 2005; Wenzel, Huang, Armstrong, Walker, & Cella, 2007). Taxanes are plant derived and considered microtubule-stabilizing agents that block mitosis in the late G2 mitotic phase of the cell cycle, inducing cell death (Lee & Swain, 2006). Paclitaxel and docetaxel, the two most widely used chemotherapy drugs in the taxane family, are effective in the treatment of many common cancers, including lung, breast, prostate, and gynecologic malignancies (Eniu, Palmieri, & Perez, 2005; Jones et al., 2005; Pazdur, Kudelka, Kavanagh, Cohen, & Raber, 1993; Seidman et al., 2008; Shimozuma et al., 2012). Despite the benefits of taxane-based chemotherapy, patients frequently experience neuropathic symptoms associated with treatment known as chemotherapy-induced peripheral neuropathy (CIPN; Almadrones, McGuire, Walczak, Florio, & Tian, 2004; Flatters & Bennett, 2006; Lee & Swain, 2006; Markman, 2003; Speck et al., 2012). Peripheral neuropathy remains a persistent, dose-limiting side effect of both paclitaxel and docetaxel (Stubblefield, McNeely, Alfano, & Mayer, 2012).

The risk of developing taxane-induced peripheral neuropathy is dependent on the drug, schedule, cumulative dose, and presence of preexisting medical conditions (Kanbayashi et al., 2010). Peripheral neuropathy is distressing to patients in that it interferes with their ability to carry out normal activities and diminishes their quality of life (Almadrones et al., 2004; Bakitas, 2007; Hile, Fitzgerald, & Studenski, 2010; Tofthagen, 2010).

Chemotherapy-induced peripheral neuropathy is a growing topic of research, but there are still limited data concerning the symptoms of taxane-induced peripheral neuropathy and its effects on quality of life. Few studies have compared CIPN symptoms caused by paclitaxel with those caused by docetaxel, although previous studies suggest that docetaxel-induced neuropathies may be less severe and occur with less frequency than paclitaxel-induced neuropathies (Rose & Smrekar, 2003; Swain & Arezzo, 2008). Therefore, the purpose of this study was to explore the prevalence, severity, distress, and timing of neuropathic symptoms in cancer patients receiving taxanes and to explore interference with usual activities.

Taxanes are believed to induce sensory and motor neuropathy by impairing axon structure and function, most specifically by inducing mitochondrial and vascular dysfunction (Polomano, Mannes, Clark, & Bennett, 2001). Upon infusion, paclitaxel induces a rapid decline in axonal mitochondrial membrane potential, spontaneous neuronal firing, and reactive oxygen species production, resulting in functionally impaired, swollen, and vacuolated axonal mitochondria in both unmyelinated and myelinated axons (Flatters & Bennett, 2006; Siau, Xiao, & Bennett, 2006). In laboratory experiments, animals treated with paclitaxel developed a reduction in the numbers of vasa nervorum (small arterioles that supply peripheral nerves), attenuated nerve blood flow, and marked endothelial cell apoptosis (Kirchmair et al., 2007). Alterations in mitochondrial dysfunction and microvascular damage are the dual mechanisms that underlie taxane-induced CIPN, resulting in neuropathic pain, hyperalgesia, and/or loss of sensation and functional ability. Symptoms vary based on drug and cumulative dose as well as individual differences.

## Paclitaxel

Sensory manifestations associated with paclitaxel-induced CIPN include distal, symmetrical sensory loss; impairments in vibration; reduced proprioception; and sensations of numbness, tingling, and burning pain in a stocking and glove pattern (Eniu, Palmieri, & Perez, 2005; Visovsky, Collins, Abbott, Aschenbrenner, & Hart, 2007). Motor and sensory neuronal loss in the lower extremities results in weakness of large lower extremity muscle groups and reduced gait performance and ability to compensate for changes in terrain (Manor & Li, 2009; Van Schie, 2008). Symptoms may develop within 24 to 72 hours of treatment and after a cumulative dose of 135–200 mg/m^2^, or after a single dose > 250 mg/m^2^ (Pazdur et al., 1993).

## Docetaxel

Docetaxel is a potent agent in inhibiting cell replication that is also associated with both sensory and motor peripheral neuropathy (Sanofi-Aventis, 2013). Signs and symptoms begin with paresthesias, numbness in the hands and feet, loss of deep tendon reflexes, and vibration sensation. Burning, tingling, loss of joint position sense, heaviness in hands and feet, weakness, and loss of ankle and knee jerk have been described as characteristic signs and symptoms associated with docetaxel-induced peripheral neuropathy. These symptoms can lead to a loss of dexterity, gait disturbances, clumsiness, pain, and disability. At cumulative doses above 600 mg, moderate to severe peripheral neuropathy can develop (Hilkens, Verweij, Vecht, Stoter, & van den Bent, 1997).

Although signs and symptoms can begin after administration of docetaxel, most symptoms develop after two to four cycles. The symptoms can improve in between cycles, with gradual progression of symptoms with subsequent cycles. In most patients, the symptoms of peripheral neuropathy resolve over a period of weeks following discontinuation of docetaxel (Hilkens et al., 1997). A phase III metastatic breast cancer study documented the onset of grade 2 or greater neuropathy occurring at a cumulative dose of 371 mg/m^2^ (Jones et al., 2005). The relationship between weekly vs. every-3-week administration of docetaxel and neuropathy is uncertain, as studies have revealed conflicting results (Di Maio et al., 2007; Rivera et al., 2008).

## Methods

**SAMPLE AND SETTING**

This cross-sectional, descriptive study included patients receiving paclitaxel or docetaxel for treatment of any type of malignancy. The sample was accrued at two sites: a large National Cancer Institute (NCI)-designated Comprehensive Cancer Center in west central Florida and a private medical oncology practice in the same geographic area. Only adult patients who could read, write, and understand English were included. Patients were excluded if they were receiving their first cycle of paclitaxel or docetaxel on the day of consent.

**INSTRUMENTS**

The Chemotherapy Induced Peripheral Neuropathy Assessment Tool (CIPNAT) contains two sets of items: symptom experience and interference items. The symptom experience items measure nine neuropathic symptoms: numbness in the hands, numbness in the feet, tingling in the hands, tingling in the feet, sensitivity to cold temperatures, nerve pain, muscle/joint aches, muscle weakness, and loss of balance. For each symptom reported, participants were asked to rate the intensity, distress, and frequency of that symptom on a 0 to 10 numeric rating scale. Higher scores on the symptom experience scale correspond with higher degrees of CIPN. Scores on the symptom experience items range from 0 to 279, with higher scores indicating more neuropathy (Tofthagen, McMillan, & Kip, 2011).

The 14 interference items ask participants to rate, on a scale of 0 (not at all interfering) to 10 (completely interfering), how much their symptoms interfere with their ability to perform specific activities: dressing, walking, picking up objects, holding onto objects, driving, working, participating in hobbies or leisure activities, exercising, engaging in sexual activity, sleeping, having relationships with others, writing, doing household chores, and enjoying life. Higher scores on the interference scale correspond with greater neuropathic interference with usual activities (poorer functional status). Scores on the interference items range from 0 to 140, with higher scores indicating more significant neuropathic interference with usual activities.

Correlations with a measure of the same or similar concept (r = .83, * p* < .001) and differences between contrasting groups (t = 7.66, * p* < .001) provided evidence of construct validity. High test-retest correlations (r = .921, * p* < .001) and Cronbach’s alpha (á = .927) provided evidence of reliability (Tofthagen, McMillan, & Kip, 2011).

**PROCEDURES**

The study was approved by the Institutional Review Board of the University of South Florida. Eligible patients were identified with the help of nurses in the infusion center. Patients meeting eligibility criteria were invited to participate during a regularly scheduled visit to the outpatient chemotherapy infusion center, and informed consent was obtained. Study participants completed the CIPNAT and a demographic data form, which included age, gender, race/ethnicity, income, education, marital status, years of formal education, and employment status. Information regarding the type of cancer, chemotherapy regimen, number of chemotherapy cycles, and cumulative dose of neurotoxic agent was obtained from the medical record (see Tables 1 and 2). The purpose of the parent study from which these data came was to evaluate reliability and validity of the CIPNAT. The method and results of that study have been described in detail in an earlier publication (Tofthagen, McMillan, & Kip, 2011).

**Table 1:**
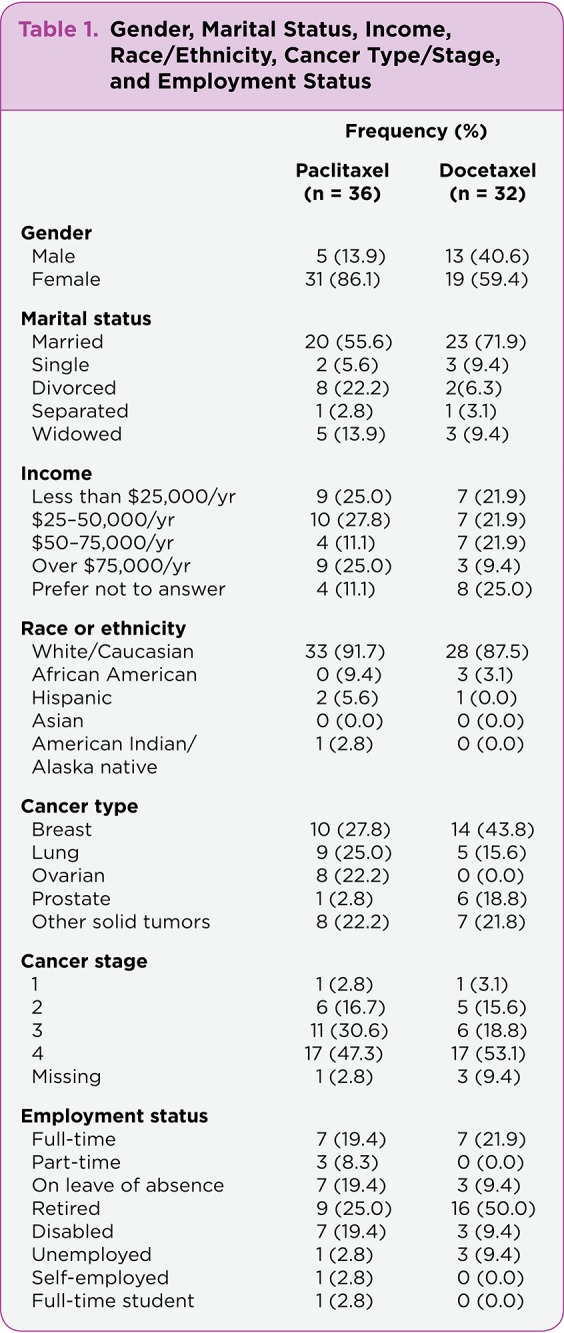
Table 1. Gender, Marital Status, Income, Race/Ethnicity, Cancer Type/Stage, and Employment Status

**Table 2:**
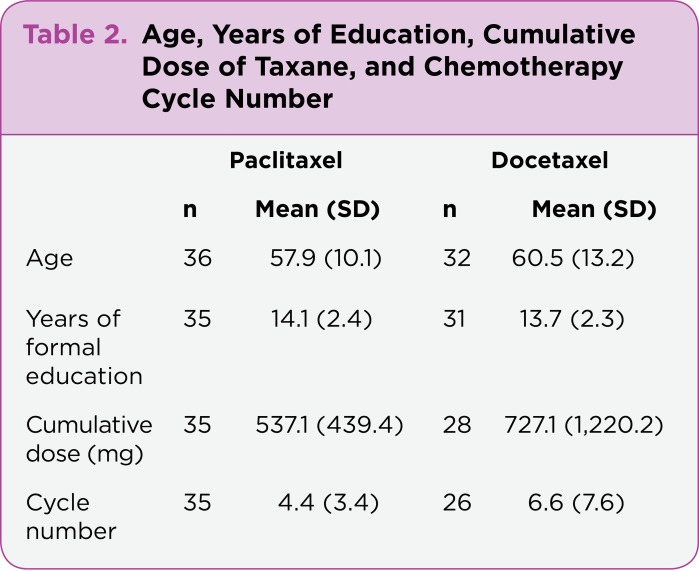
Table 2. Age, Years of Education, Cumulative Dose of Taxane, and Chemotherapy Cycle Number

**DATA ANALYSIS**

Data were analyzed using descriptive statistics. Mean severity, distress, and frequency scores for each symptom were calculated using data from participants who reported having that symptom. T tests were used to determine differences in prevalence of individual symptoms and total scores on the CIPNAT between persons receiving paclitaxel and those receiving docetaxel.

## Results

The sample of 68 patients was almost evenly divided between those who were receiving paclitaxel (n = 36) and those receiving docetaxel (n = 32). More women than men participated in the study. The majority of participants were married, Caucasian, and from a variety of income categories. Most had breast, lung, ovarian, or prostate cancer in advanced stages. Few were employed. Participants ranged in age from 24 to 80 and on average had 2 years of post–high-school education (see Table 2). Persons receiving docetaxel were slightly older and had received higher cumulative doses and more cycles on average than those receiving paclitaxel. A total of 19.4% (n = 7) of participants who were receiving paclitaxel had been previously treated with docetaxel and 11.1% (n = 4) had been previously treated with cisplatin, another neurotoxic chemotherapy drug. None of the participants receiving docetaxel had received paclitaxel, but 9.4% (n = 3) had previously received cisplatin.

Those receiving paclitaxel were predominantly women with breast, lung, or ovarian cancer. More males were receiving docetaxel, which may be preferred over paclitaxel for treatment of metastatic prostate cancer (Armstrong & Carducci, 2005), although the majority of those receiving docetaxel were being treated for breast or lung cancer. The majority of participants in both groups had stage III/IV disease.

**NEUROPATHIC SYMPTOMS**

Total scores on the symptom experience items on the CIPNAT for patients receiving paclitaxel ranged from 6 to 202 (mean = 90.2, standard deviation [SD] = 57.8). Total scores for patients receiving docetaxel ranged from 19 to 188 (n = 86.0, SD = 47.8). All patients reported developing at least one of nine neuropathic symptoms since starting chemotherapy. Participants receiving paclitaxel reported an average of 4.3 neuropathic symptoms (SD = 2.1), and patients receiving docetaxel reported an average of 4.8 symptoms (SD = 1.9). The most commonly occurring symptoms in those receiving paclitaxel were muscle or joint aches, numb feet, and trouble with balance (see Table 3). In those receiving docetaxel, the most commonly occurring symptoms were muscle or joint aches, weakness in the arms or legs, trouble with balance, and numb hands (see Table 4). No statistically significant differences in scores on the CIPNAT symptom experience items, number of symptoms, or occurrence of individual neuropathic symptoms were found between patients receiving paclitaxel and those receiving docetaxel.

**Table 3:**
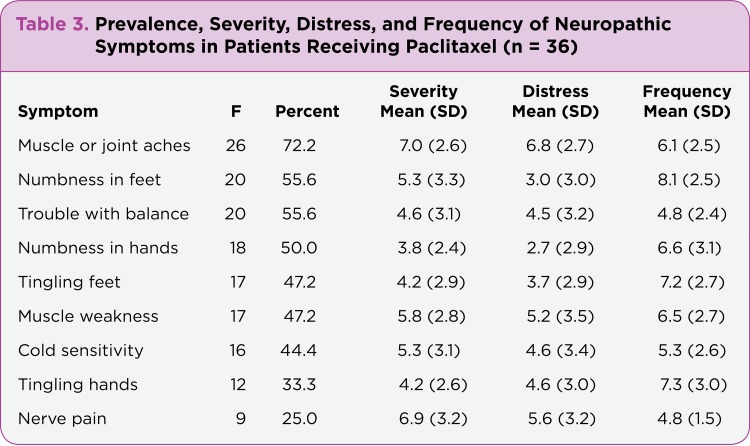
Table 3. Prevalence, Severity, Distress, and Frequency of Neuropathic Symptoms in Patients Receiving Paclitaxel (n = 36)

**Table 4:**
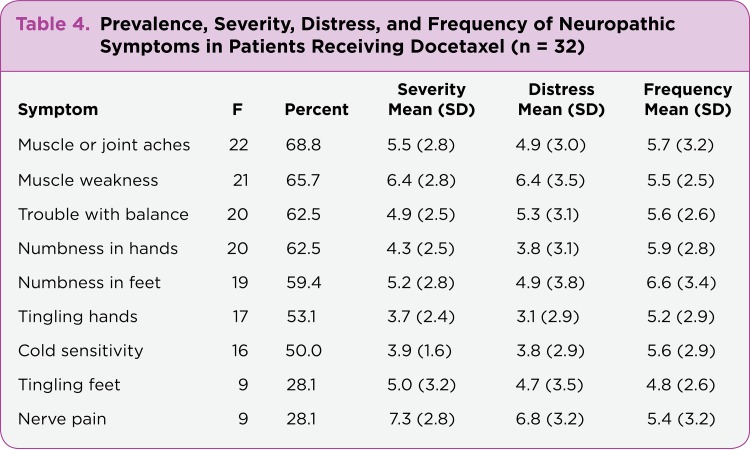
Table 4. Prevalence, Severity, Distress, and Frequency of Neuropathic Symptoms in Patients Receiving Docetaxel (n = 32)

**NUMBNESS AND TINGLING**

Participants reported varying degrees of numbness in the hands and feet. The majority of participants with numbness in the hands, receiving paclitaxel or docetaxel, reported that the numbness was confined to the fingertips or fingertips and fingers. The time of day when numbness or tingling in the hands was considered the worst varied widely between both groups (see Table 5). Among participants who reported numbness or tingling in the hands that was more severe after chemotherapy, members of both groups reported that it was most severe for 1 to 10 days.

**Table 5:**
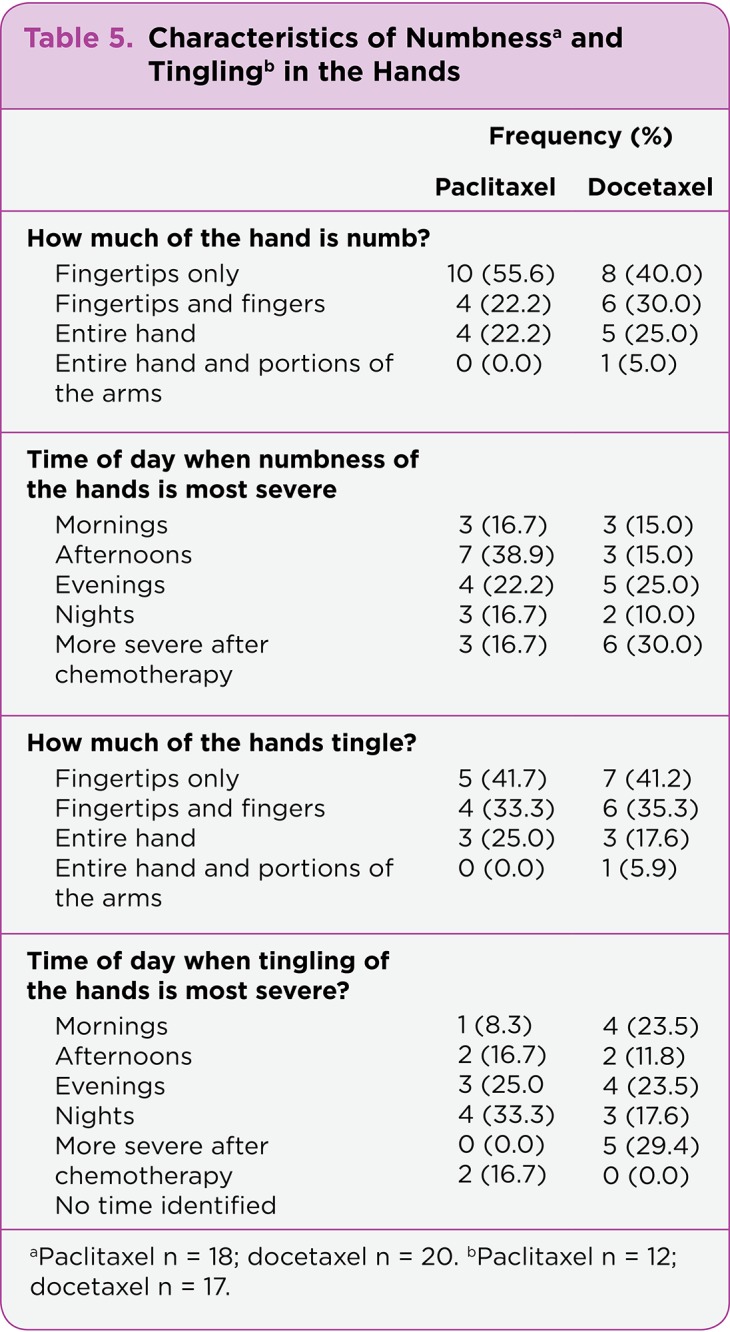
Table 5. Characteristics of Numbness and Tingling in the Hands

The majority of participants with numbness in the feet receiving paclitaxel reported that the numbness affected the toes only or the toes and balls of the feet, whereas the majority of those receiving docetaxel reported that more of the foot and sometimes parts of the legs were affected (see Table 6). The majority of participants in both groups who reported tingling in the feet reported that the tingling was not confined to the toes and balls of the feet but also involved the soles of the feet, the entire foot, or the entire foot and parts of the legs. The majority of participants with numbness or tingling in the feet reported that the numbness was most severe in the evening or at night. In those who reported more severe numbness or tingling in the feet immediately following chemotherapy, the range was from 1 to 14 days.

**Table 6:**
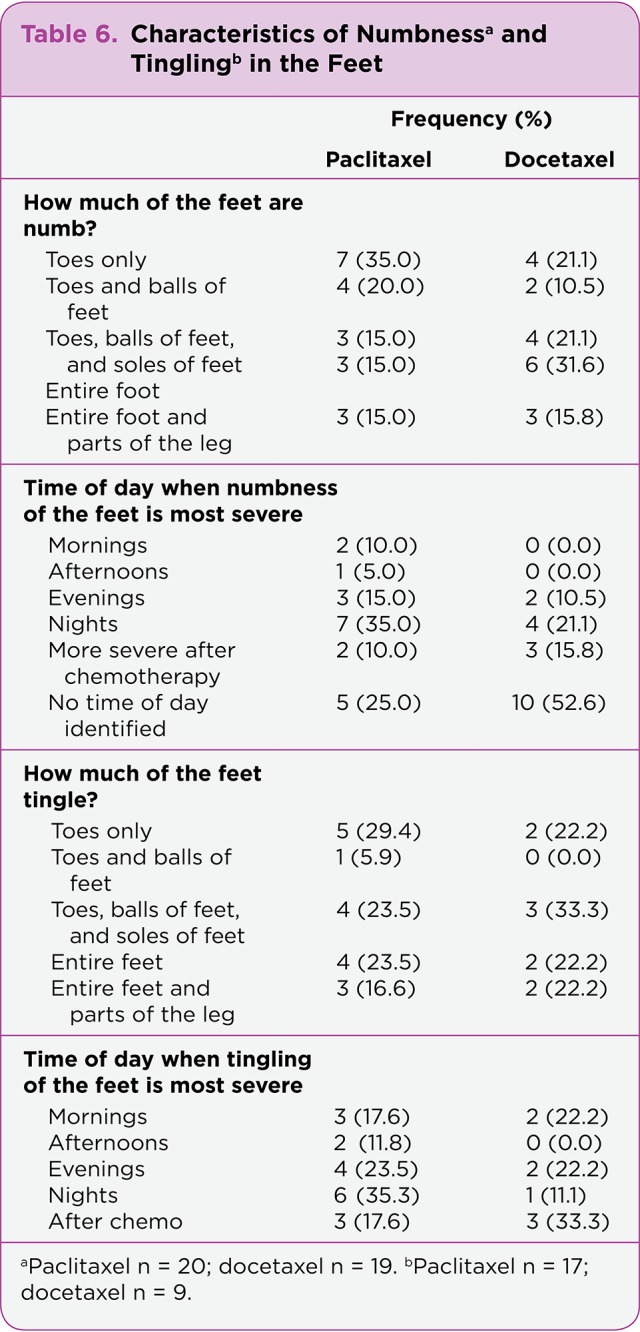
Table 6. Characteristics of Numbness and Tingling in the Feet

**COLD SENSITIVITY**

The majority of participants with cold sensitivity in both groups reported that their entire body, upper extremities, and lower extremities were affected (see Table 7). The few participants who reported that it was worse after chemotherapy said it was most severe for 1 day following chemotherapy. The rest of the participants did not report a noticeable difference in cold sensitivity following chemotherapy.

**Table 7:**
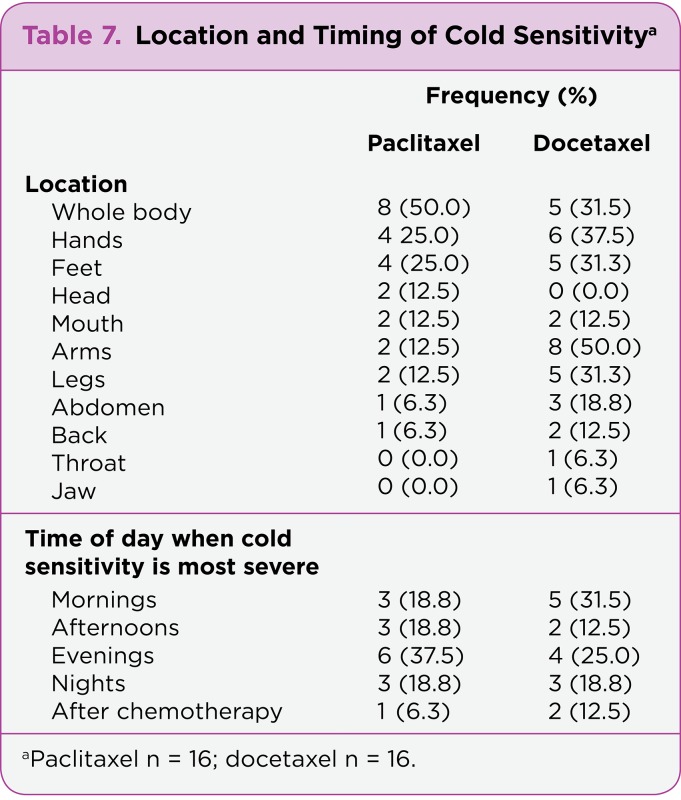
Table 7. Location and Timing of Cold Sensitivity

**NERVE PAIN**

Those who reported nerve pain most often reported that the pain occurred in the legs, feet, and hands (see Table 8). Persons who noted a specific anatomic location outside of the upper or lower extremities indicated the presence of nerve pain in the chest (n = 3), back (n = 1), side (n = 1), shoulders (n = 1), and axilla (n = 1). A variety of terms were used to characterize nerve pain. No pattern was noted in reference to the time of day when nerve pain was most severe. Among those who reported more nerve pain after chemotherapy, the most severe nerve pain occurred for 5 to 14 days.

**Table 8:**
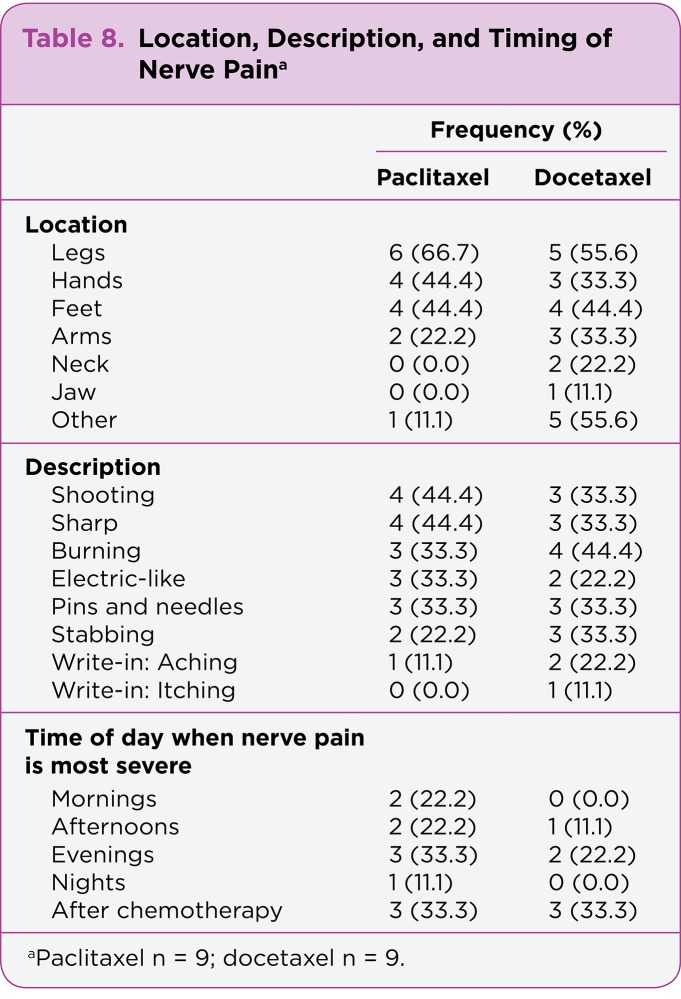
Table 8. Location, Description, and Timing of Nerve Pain

**MUSCLE/JOINT ACHES**

Muscle or joint aches were reported by the majority of participants regardless of chemotherapy agent. Muscle aches were more frequently reported in the paclitaxel group, and joint aches were more frequently reported in the docetaxel group. Aching in the legs was most frequently reported in both groups (see Table 9). Participants receiving paclitaxel reported muscle or joint aches to be most severe immediately following chemotherapy. Patients who reported more severe muscle/joint aches after chemotherapy reported that it lasted 1 to 16 days.

**Table 9:**
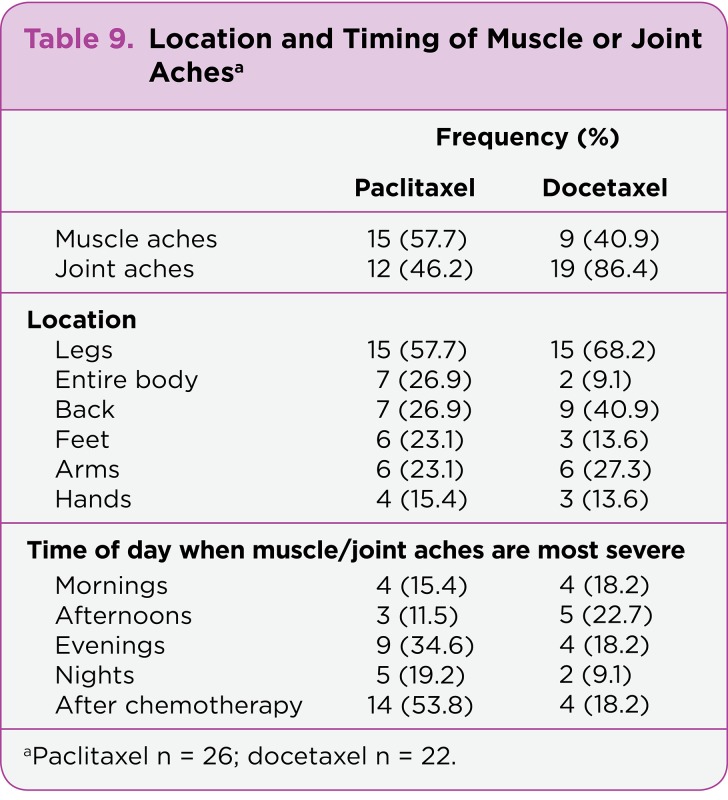
Table 9. Location and Timing of Muscle or Joint Aches

**MUSCLE WEAKNESS AND LOSS OF BALANCE**

Among participants who reported muscle weakness, weakness in the legs was almost unanimously reported (see Table 10). Again, participants were allowed to choose as many anatomical locations as applied. No pattern of time of day that muscle weakness was most severe emerged. Participants receiving paclitaxel tended to report muscle weakness being most severe after chemotherapy as compared to participants receiving docetaxel. Severe muscle weakness was reported to last 2 to 14 days.

**Table 10:**
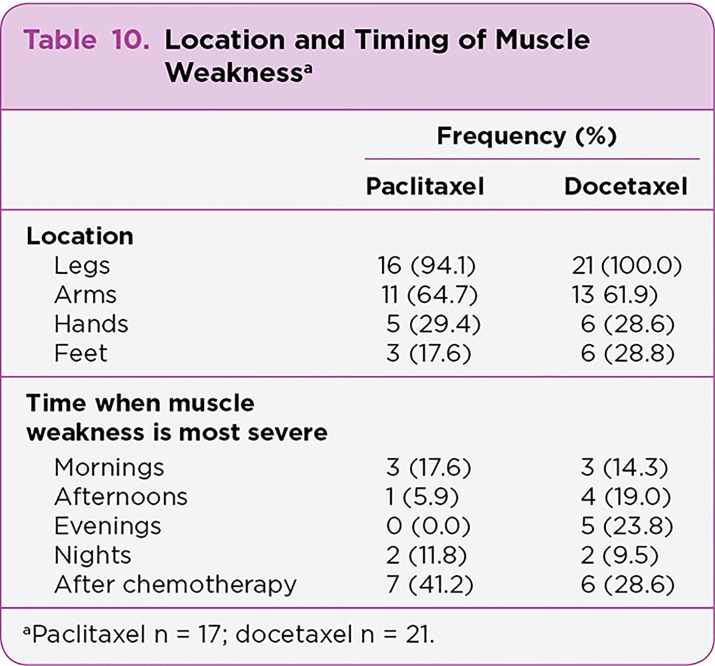
Table 10. Location and Timing of Muscle Weakness

Loss of balance was reported by over 50% of participants receiving paclitaxel or docetaxel. Those receiving paclitaxel reported that loss of balance was worse following chemotherapy and occurred more often as compared to those receiving docetaxel. Participants receiving docetaxel more often reported loss of balance to be most severe in the morning or afternoon (see Table 11). Participants reported loss of balance to be most severe 2 to 14 days following chemotherapy.

**Table 11:**
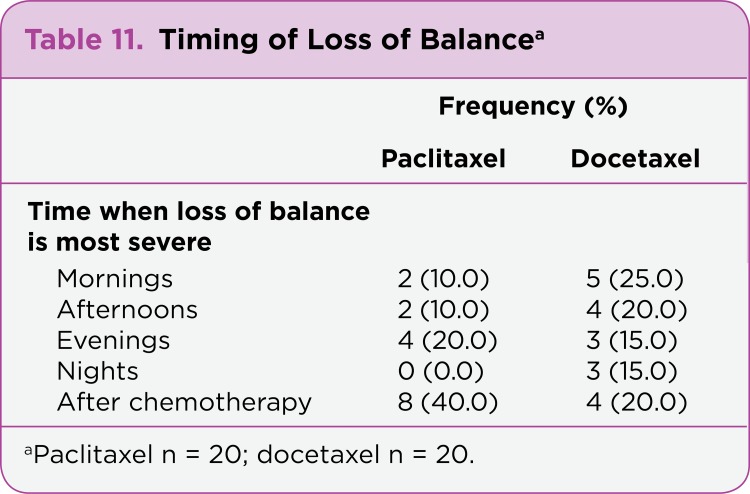
Table 11. Timing of Loss of Balance

**SEVERITY, DISTRESS, AND FREQUENCY: PACLITAXEL**

Mean severity of symptoms was 5.3 (SD = 2.4) on a scale of 0 to 10 for participants receiving paclitaxel. Participants rated muscle/joint aches as the most severe symptom, followed by nerve pain, then muscle weakness (Table 3). Mean symptom distress was 4.6 (SD = 2.3) on a scale of 0 to 10. Participants were most distressed by muscle/joint aches, followed by nerve pain, then muscle weakness. Mean symptom frequency was 6.1 (SD = 2.0), meaning that patients experienced symptoms once a day on average. Numbness in the feet was the most constant symptom, followed by tingling hands, tingling feet, and numb hands.

**SEVERITY, DISTRESS, AND FREQUENCY: DOCETAXEL**

Mean severity of symptoms was 5.0 (SD = 2.0) for participants receiving docetaxel. Participants rated nerve pain as the most severe symptom, followed by muscle weakness, then muscle or joint aches (Table 4). Mean symptom distress was 4.6 (SD = 2.5) on a scale of 0 to 10. Participants were most distressed by nerve pain, followed by muscle weakness, then trouble with balance. Mean symptom frequency was 5.8 (SD = 2.1). Numbness in the feet was the most constant symptom, followed by numbness in the hands, and muscle/joint aches.

**INTERFERENCE WITH ACTIVITIES**

Participants receiving paclitaxel reported that neuropathic symptoms interfered with a mean of 7.3 (SD = 4.1) of 14 activities. Among participants receiving paclitaxel, neuropathic symptoms most often interfered with sleep, exercise, and household chores (see Table 12). Work, driving, and exercise were the activities that were most significantly affected, meaning they had the most difficulty completing these activities. Participants receiving docetaxel reported that neuropathic symptoms interfered with a mean of 7.1 (SD = 4.1) of 14 activities. Among participants receiving docetaxel, neuropathic symptoms most often interfered with enjoyment of life, chores, and walking (see Table 12). Chores, sexual activity, and enjoyment of life were the activities that were most significantly affected.

**Table 12:**
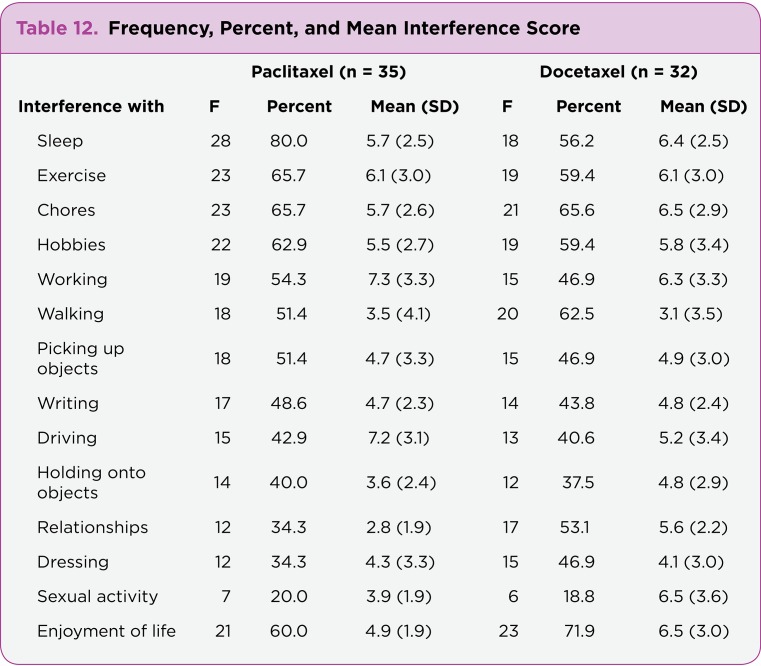
Table 12. Frequency, Percent, and Mean Interference Score

## Discussion

The results of this study highlight the presence of sensorimotor symptoms in persons receiving both paclitaxel and docetaxel. In both chemotherapy regimens, neuropathic symptoms caused significant interference with activities and enjoyment of life.

Motor symptoms, including muscle or joint aches, muscle weakness, and loss of balance, were the most prevalent symptoms reported by persons receiving docetaxel. Motor symptoms were also frequently reported in those receiving paclitaxel. Numbness in the feet was the most prevalent sensory symptom reported in both groups and may contribute to loss of balance, falls, and other injuries (Tofthagen, Overcash, & Kip, 2012). On average, members of both groups reported more than four neuropathic symptoms. Chemotherapy-induced peripheral neuropathy is a complex phenomenon, and symptoms vary widely based on neurotoxic drug(s), cumulative dose, comorbidities such as diabetes, and other factors (Argyriou et al., 2006; Wampler et al., 2007).

**NUMBNESS AND TINGLING**

In this sample, foot symptoms were more problematic than hand symptoms. Numbness and tingling in the feet may lead to falls, blisters, burns, and other injuries (Tofthagen, Overcash, & Kip, 2012). Interventions aimed at improving safety should be an important component of education for patients with CIPN (Wickham, 2007). Numbness and tingling of the feet were reported to be worst at night. It may be that these symptoms are actually more noticeable at night when someone is trying to rest, but further studies aimed at alleviating symptoms and improving sleep quality are needed.

**COLD SENSITIVITY**

Persons receiving paclitaxel tended to report more generalized cold sensitivity, while those receiving docetaxel reported that specific body parts—mainly upper and lower extremities—were more sensitive to cold temperatures. Cold sensitivity may tend to be more severe in the evenings. Since this study was conducted during the autumn season in Florida when temperatures are generally warm at all times, climate probably had little bearing on these results. Adding extra layers of clothing, taking warm showers or baths, and adjusting the thermostat to a warmer temperature may be helpful for patients who are experiencing cold sensitivity.

In relationship to CIPN, cold sensitivity is usually associated with the acute neurotoxic effects of oxaliplatin and has not been previously examined as a neurotoxic side effect of other chemotherapy drugs. Hair and weight loss and decreases in activity may also contribute to cold sensitivity. Additional research into acute neurotoxicity and associated symptom clusters is needed.

**NERVE PAIN**

Neuropathic pain, though not as prevalent as other neuropathic symptoms, was reported to be among the most severe and distressing symptoms and was the most severe and distressing symptom in persons receiving docetaxel. While painless symptoms like numbness and loss of balance may be more prevalent, the results of this study indicate that neuropathic pain remains problematic for persons receiving taxanes. Many treatment options for neuropathic pain are available, including antiseizure medications, antidepressants, and topical analgesia. An aggressive approach to management of neuropathic pain may improve physical performance and emotional well-being (Moulin et al., 2007; Smith, Whedon, & Bookbinder, 2002; Smith et al., 2011).

The results of this study also support the findings of previous research, indicating that sharp, stabbing, shooting, burning, electric-like, and pinsandneedles are descriptors commonly used by persons with neuropathic pain (Boureau, Doubrere, & Luu, 1990; Mystakidou et al., 2007). In this study, aching was also used as a descriptor. Thorough pain assessment includes a description of the pain. When patients use these words to describe their pain, a neuropathic origin should be suspected, particularly in patients receiving neurotoxic chemotherapy drugs like taxanes; aggressive pain management techniques should be instituted.

**MUSCLE/JOINT ACHES**

Muscle/joint aches were the most prevalent symptom in persons receiving paclitaxel and docetaxel and the most severe and distressing symptom among persons receiving paclitaxel. Particularly in persons receiving paclitaxel, these symptoms tended to be more severe following chemotherapy. Analgesics should be prescribed on an as-needed basis for these patients and satisfaction with pain control assessed regularly. Muscle/joint aches have previously been described as a side effect of paclitaxel and have primarily been viewed as an acute side effect following chemotherapy (Markman, 2003; Nguyen & Lawrence, 2004). This study indicated that this is also a prevalent and distressing symptom for persons receiving docetaxel, although it is possible that muscle/joint aches have an outside cause, such as treatment with white blood cell growth factors (filgrastim [Neupogen]) or comorbidities, including arthritis. Future research should examine differences in muscle/joint aches between people receiving paclitaxel and docetaxel who do not develop neuropathy. Comorbid conditions also need further exploration in relation to the development of neuropathic symptoms in cancer and cancer treatments.

**MUSCLE WEAKNESS AND LOSS OF BALANCE**

Muscle weakness and loss of balance were reported by approximately half of the participants receiving both medications. Participants reported that weakness in the legs (and to a lesser degree the arms) was more of a problem than weakness in the hands and feet. Muscle strength in the arms is necessary to lift, carry, push, and pull. Muscle strength in the legs is necessary to maintain balance and mobility. Comorbidities, advancing stages of malignancy, frailty, and advancing age are all factors that may also contribute to muscle weakness and loss of balance and may have influenced the outcome of this study in some way. Future studies should examine risk factors for falls in persons with CIPN. More studies of interventions designed to improve strength and balance in patients with CIPN are needed.

**INTERFERENCE WITH ACTIVITIES**

The participants in this study reported that their CIPN greatly interfered with many important daily functions and aspects of quality of life. Clinicians and researchers need to continue to work together to find ways to minimize both the symptoms of CIPN and their impact on physical functioning and quality of life.

## Limitations

This study sample was collected from one geographic location with a predominantly female, Caucasian population and may not be generalizable to other populations. Studies involving a more ethnically and racially diverse sample are needed. In this study, more participants receiving paclitaxel had received other neurotoxic chemotherapy drugs in the past, which may have also affected the results of this study. While it would seem likely that previous treatment with neurotoxic chemotherapy would result in increased neurotoxicity and more interference with activities, this was not substantiated in the study.

## Conclusions

Patients receiving paclitaxel and docetaxel experienced similar symptoms of CIPN and similar neuropathic interference with activities. Muscle or joint aches were the most prevalent symptoms and numbness in the feet was the most frequent or constant symptom among both patients receiving paclitaxel and those receiving docetaxel. Painful symptoms including neuropathic pain, and muscle/joint aches were reported by patients to be more severe and distressing, while painless symptoms including numbness and tingling and loss of balance occurred with greater frequency. A continued clinical focus on relief of neuropathic pain and enhancement of physical performance is warranted as researchers investigate ways to prevent CIPN.
